# Bioinformatics analysis and experimental validation revealed that Paeoniflorigenone effectively mitigates cerebral ischemic stroke by suppressing oxidative stress and inflammation

**DOI:** 10.1038/s41598-024-55041-5

**Published:** 2024-03-07

**Authors:** Zhiyan Wu, Xingrong Tang

**Affiliations:** 1 Department of Preventive Treatment, Dongguan Humen Hosipital of Traditional Chinese Medicine, Building No.375, Jienan lu, Dongguan, 523900 Guangdong China; 2 Department of Science and Education, Jiangmen Wuyi Hospital of Traditional Chinese Medicine, Building No.30, Huayuandong lu, Jiangmen, 529000 Guangdong China

**Keywords:** Paeoniflorigenone, Bioinformatics analysis, Reactive oxygen species, Cerebral ischemia and reperfusion, Inflammation, Computational biology and bioinformatics, Pharmacology

## Abstract

Inflammation and oxidative stress are becoming more recognized as risk factors for ischemic stroke. Paeoniflorigenone (PA) has diverse pharmacological effects that include anti-inflammatory and antioxidant properties. However, the specific mechanisms by which PA affects cerebral ischemic stroke have not been studied. Our objective was to investigate the potential targets and mechanisms of PA in preventing cerebral ischemic stroke. We obtained the potential targets of PA from the SwissTargetPrediction, Super-PRED, and SEA Search Server databases. The GSE97537 dataset was utilized to identify gene targets related to ischemic stroke. The overlapping targets were imported into the STRING database to construct a protein–protein interaction network, and enrichment analyses were conducted using R software. Rats were pretreated with PA for three weeks before undergoing MCAO and reperfusion. H&E staining, ELISA, and qRT-PCR analyses were then performed to explore the potential mechanisms of PA. In the study, we identified 439 potential targets for PA and 1206 potential targets for ischemic stroke. Out of these, there were 71 common targets, which were found to be primarily associated with pathways related to oxidative stress and inflammation. The results from animal experiments showed that PA was able to improve nerve function and reduce inflammatory cytokines and oxidative stress in the MCAO-induced ischemic stroke model. Additionally, the expression of core genes in the MCAO + HPA group was significantly lower compared to the MCAO group. Our study revealed that the potential mechanisms by which PA prevents ischemic stroke involve oxidative stress and inflammation. These findings provide important theoretical guidance for the clinical use of PA in preventing and managing ischemic stroke.

## Introduction

Stroke is a common clinical syndrome characterized by three main pathological subtypes: subarachnoid hemorrhage, intracerebral hemorrhage, and ischemic stroke^1^. It is a primary cause of disability and ranks as the second leading cause of death in adults globally. This places a significant financial burden on both families and countries^2^. New research findings indicate that over 80% of stroke incidents are ischemic strokes, which occur due to blockage in the cerebral arteries^3–5^. Insufficient blood supply to the brain, usually caused by the blockage of blood vessels, can trigger an ischemic stroke. This condition can result in serious neurological damage and brain injury^6^. Recent research has revealed that although numerous drugs have been subjected to clinical trials or are currently being evaluated in preclinical studies for the treatment of ischemic stroke, there is still a scarcity of clinically approved and effective neuroprotective agents^7^. The development of drugs for the treatment of ischemic stroke has increasingly relied on the exploration of natural products, which have emerged as crucial resources for the development of new therapeutic agents. These natural products have demonstrated significant neuroprotective potential in preclinical studies, further highlighting their importance in the pursuit of effective treatments for ischemic stroke^8^. Therefore, the development of therapy utilizing phytochemicals holds immense clinical significance in improving the outcomes of patients with ischemic stroke.

The medicinal properties of Paonia suffruticosa Andr., a member of the Paeoniaceae family, have long been recognized and used to treat various health conditions. Its root bark has been traditionally employed in the treatment of reproductive disorders cancer, inflammation, heart problems, and vascular issues^9–11^. Numerous chemical compounds have been extracted from P. suffruticosa, including monoterpene glycosides, stilbenes, tannins, and phenols^10^. Of particular interest is paeoniflorigenone (PA), which demonstrates a diverse array of bioactive properties. Paeo-mediated biological mechanisms have been discovered to demonstrate anti-oral cancer effects by targeting the PI3K/AKT/mTOR/p70S6K signaling pathway^12^. PA protects neuronal cells against cytotoxicity induced by H_2_O_2_ and also inhibits nitric oxide production through the activation of lipopolysaccharide-activated microglia^13,14^. Moreover, PA has the potential to enhance blood flow by inhibiting the aggregation of platelets and/or the coagulation of blood^15^. Nevertheless, the specific pharmacological effects and mechanisms of PA on cerebral ischemic stroke remain unclear.

The rapid advancement of big data, networks, computers, and bioinformatics has revolutionized the exploration of traditional Chinese medicine, leading to substantial achievements^16–18^. Furthermore, the emergence of RNA-sequencing and microarray technologies has significantly expanded our knowledge of pathological mechanisms through the analysis of mRNA expression profiles in human diseases. By utilizing bioinformatics analysis to compare differentially expressed genes (DEGs) between normal and disease groups, we can accurately, efficiently, and systematically identify core genes and potential therapeutic targets^19,20^. By employing bioinformatics analysis, researchers have identified NCF4, IL7R, and SLAMF1 as promising therapeutic targets for enhancing histological recovery following an ischemic stroke^21^. Furthermore, a recent study utilizing bioinformatics analysis has discovered six central mRNAs (Cxcl1, Socs3, Mmp9, Stat3, Il-1β, and Ptgs2) that play significant roles in the development of ischemic stroke^22^.

**Figure 1:**
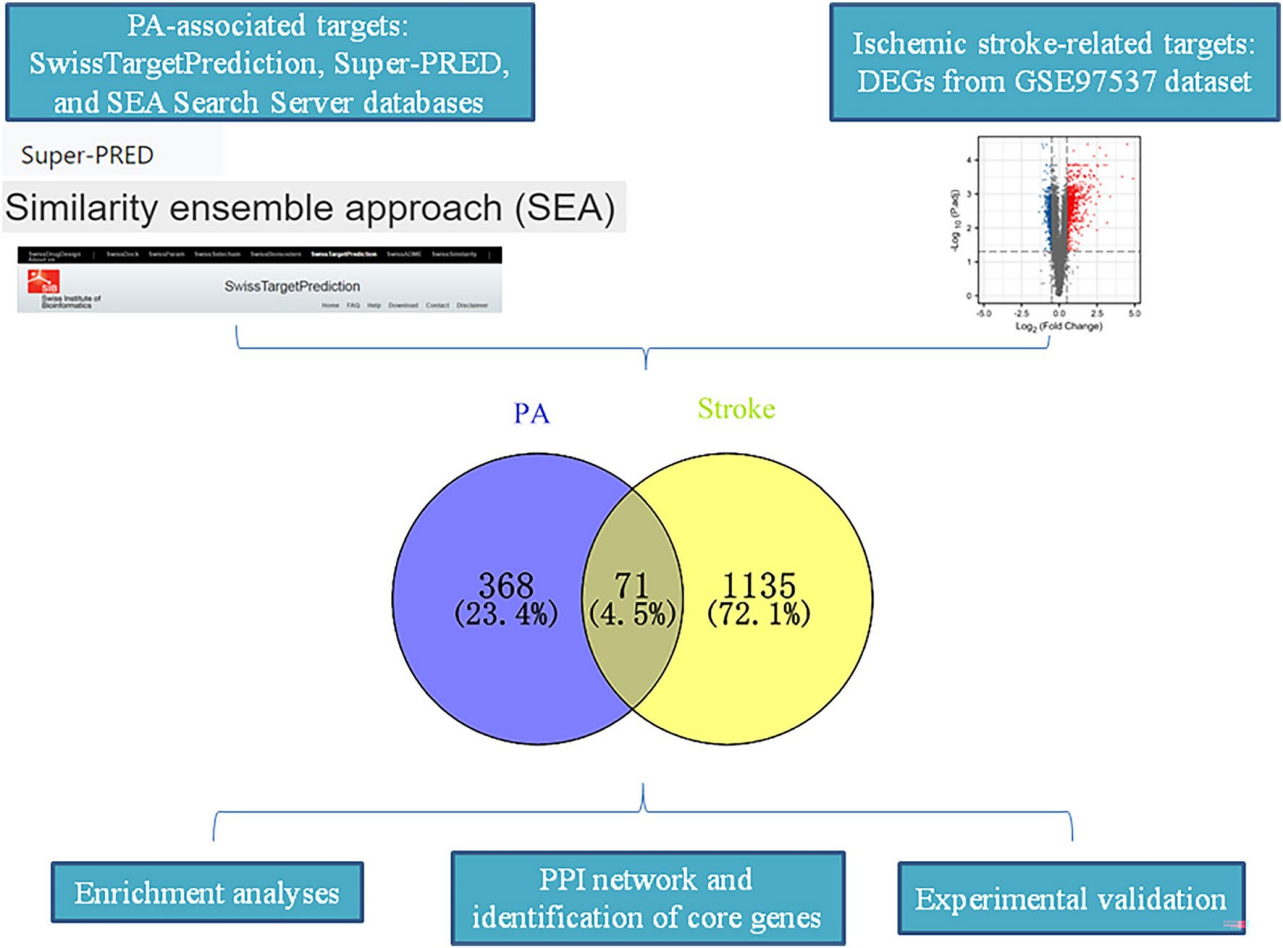
The flow diagram for identifying the anti-ischemic stroke mechanisms of PA based on bioinformatics analysis and experimental validation.

Considering the intricate mechanism of PA and the multifaceted pathogenesis of ischemic stroke, there is a need to delve deeper into the therapeutic mechanisms of PA in combating this condition. To shed light on these matters, we conducted a comprehensive study utilizing a combination of bioinformatics analysis and in vivo experiments to explore the potential mechanisms by which PA may contribute to the prevention of ischemic stroke. We utilized various databases and conducted functional enrichment analysis to identify and predict the potential targets and pathways associated with PA in the context of ischemic stroke. Additionally, we conducted animal experiments to validate the results obtained from the bioinformatics analysis. The flow diagram illustrating the methodology employed in this study is depicted in Fig. 1. Through our research, we have made initial strides in understanding the potential mechanism by which PA may help prevent ischemic stroke. Our findings serve as a theoretical basis that could inform the clinical application of PA in the future.

## Methods and materials

### Collection of PA-associated targets

SwissTargetPrediction is an online tool, that aims to predict the most likely protein targets of small molecules based on the combination of 2D and 3D similarity measures with known ligands^23^. The SwissTargetPrediction database was applied to screen the main active ingredients’ targets in PA. Furthermore, we utilized two additional tools, namely Super-PRED (https://prediction.charite.de/index.php) and SEA Search Server (https://sea.bkslab.org/), to gather PA-related targets. The targets obtained from these three databases were then merged and any duplicates were removed, resulting in a final set of PA-related targets.

### Screening of ischemic stroke-related targets in the GEO database

The gene expression profiles of the GSE97537 dataset were obtained from the GEO database. In this dataset, rats were divided into two groups: the middle cerebral artery occlusion (MCAO) group (n = 7) and the sham operation group (n = 5). The raw data of GSE97537 were processed using R software (version 3.6.1). We utilized the "affy" package to perform quartile data normalization and background correction for the microarray data. To identify the differentially expressed genes (DEGs) between the MCAO and sham groups, we employed the limma package. The screening parameters used were p.adj < 0.05 and absolute log2 fold change > 0.5^24^. We utilized the ggplot2 package in R software to create a volcano plot of the differentially expressed genes (DEGs). Additionally, the ComplexHeatmap package in R software was used to generate a heatmap to visualize the expression patterns of these DEGs.

### Functional enrichment analysis and protein–protein interaction (PPI) network construction

We utilized the Venn tool (https://bioinfogp.cnb.csic.es/tools/venny/index.html) to identify the overlapping genes between PA and DEGs. To investigate the potential signaling pathways involved in the treatment of ischemic stroke using PA, we conducted an enrichment analysis of the Kyoto Encyclopedia of Genes and Genomes (KEGG) and Biological Process (BP) using the Bioconductor and cluster profile packages in R software. A p-value threshold of < 0.05 was used for screening. The overlapped genes were then uploaded to the String database (https://www.string-db.org/), selecting Rattus norvegicus as the species, and a confidence score > 0.4 was set to obtain the TSV format file^25^. Next, we visualized the protein–protein interaction (PPI) network of the overlapped genes using the Cytoscape software (version 3.8.0)^26^. To identify the core targets from the PPI network, we utilized the cytoHubba plugin available in the Cytoscape software. This plugin uses the degree value as a measure to determine the importance of nodes in the network.

### Animals

The study was conducted using male and female Wistar rats, aged 8–10 weeks, which were obtained from the Experimental Animal Center of Guangdong Province. The rats were kept in a controlled environment with an ambient temperature of 20–22 °C, humidity ranging from 45 and 55%, and a 12-h dark/light cycle. They were provided with free access to water and a standard chow diet. All animal experiments were carried out following the principles stated in the Declaration of Helsinki and were approved by the Medical Ethical Committee of Jiangmen Wuyi Hospital of Traditional Chinese Medicine (approval number: 2022064A).

### Experimental protocol

A total of 144 rats were randomly assigned to one of four groups, with 36 rats in each group: the sham group, the MCAO group, the MCAO + LPA group, and the MCAO + HPA group. In the MCAO + LPA group, the MCAO rats were orally administered a low dose of PA (LPA, 15 mg/kg) once a day. In the MCAO + HPA group, the MCAO rats were orally administered a high dose of PA (HPA, 30 mg/kg) once a day. The PA used in the study was purchased from Shanghai Yuanye Bio-Technology Co., Ltd (Shanghai, China) and was dissolved in normal saline. The sham group and the MCAO group received an equal volume of normal saline by oral administration once a day for three weeks (Fig. S1). The MCAO model was established based on a previously conducted study. In this model, the rats underwent 2 h of MCAO followed by 22 h of reperfusion^27^. After the rats were anesthetized, a monofilament was inserted into the left internal carotid artery and moved forward to block the middle cerebral artery for 2 h. Following the 2-h occlusion, the animals were briefly re-anesthetized, and the monofilament was removed to commence the reperfusion studies.

### Assessment of neurological function

After 2 h of MCAO followed by 22 h of reperfusion, we evaluated the neurological deficits using a scoring system that was based on the following criteria: a score of 0 indicated no observed neurological deficit; a score of 1 indicated the inability to extend the forepaw; a score of 2 indicated circling towards the contralateral side; a score of 3 indicated falling to the opposite side; a score of 4 indicated the inability to walk spontaneously and signs of depression. These scores were assigned to assess and quantify the severity of the neurological impairments observed in the rats after the reperfusion period^28^.

### Brain water content

Rats were anesthetized, the brain tissues were collected quickly and wet weight was recorded. After the brain tissues were dried, the dry weight was recorded again. The brain water content was determined based on the following formula: [(wet weight-dry weight) / wet weight × 100%].

### Tetrazolium chloride (TTC) staining

Tissue slices from the brain were prepared at a thickness of 2 mm. These slices underwent a staining process using a 2% solution of TTC at a temperature of 37 °C for 30 min, followed by fixation in a 4% paraformaldehyde solution at 4 °C for the duration of one night. Afterward, the slices were imaged, and the extent of the brain infarction was quantified and examined using the Image J software.

### Hematoxylin–eosin (H&E) staining

The rats were first anesthetized and then immediately sacrificed. A portion of their brain tissues was quickly collected and stored at − 20 ℃ for future analysis. Another portion of the brain tissues was placed in a buffer solution containing 4% formaldehyde fixative and left overnight for fixation. Subsequently, the fixed brain tissues were embedded in paraffin and sliced into sections that were approximately 5 µm thick. These sections were then stained using H&E staining, a common method used to visualize cellular structures in tissue samples. Finally, images of the stained slides were captured using an optical microscope. This allowed for further examination and analysis of the cellular changes and morphology within the brain tissues.

### Cell experiments

The PC12 cells used in this study were provided by the Chinese Academy of Medical Sciences. To cultivate the PC12 cells, a glass flask containing DMEM enriched with 10% FBS was prepared and placed inside an incubator adjusted to a temperature of 37 °C in 95% O_2_ and 5% CO_2_. The growth medium was refreshed every day, and when the cells reached 80–90% confluency, they were subcultured. For the experiments, only cells in the logarithmic growth phase were utilized.

To simulate the damage caused by ischemia/reperfusion injury in the brain, we established an in vitro model of oxygen–glucose deprivation/reperfusion (OGD/R) following previously described methods^29^. Initially, PC12 cells were cultured in a glucose-free medium and placed in an incubator at a temperature of 37 °C with an atmosphere consisting of 5% CO_2_, 94% N_2_, and 1% O_2_. Following two hours of oxygen–glucose deprivation (OGD), PC cells underwent a 24-h reperfusion period in a normal incubator, receiving a complete medium for nourishment. In contrast, control cell cultures were left undisturbed under normal conditions, without oxygen or glucose deprivation. In the drug groups, cells were first treated with varying concentrations of PA for 6 h, specifically 2.5 µg/mL, 5 µg/mL, and 10 µg/mL, before being subjected to OGD/R injury. The cell viability was assessed by employing a CCK-8 assay following the guidelines provided by the manufacturer. Inflammatory cytokines (IL-6 and IL-1β) and markers of oxidative stress (SOD and GSH-Px) levels were determined by harvesting culture media following OGD/R injury.

### Determination of inflammatory cytokines and oxidative stress markers

To begin with, the brain tissues were washed thoroughly with ice-cold normal saline to remove any external contaminants. Next, the tissues were prepared as a homogenate using a homogenizer to ensure a consistent and uniform sample. After homogenization, the samples were centrifuged at 5000 *g* (4 °C) for 10 min. This process helped to separate the solid components of the tissue from the liquid. The resulting supernatant was collected for further analysis. For the analysis, we utilized ELISA kits to measure the levels of inflammatory cytokines and oxidative stress markers in the collected supernatant. Commercial kits from Nanjing Jiancheng Bioengineering Institute in Nanjing, China were used for this purpose. These kits are designed to accurately measure the levels of total protein, IL-6, IL-1β, SOD, and GSH-Px, and the measurements were performed following the protocols provided by the manufacturer.

### Quantification of core gene expression

We utilized TRIzol reagent (Invitrogen, USA) to extract the total RNA from brain tissues. Subsequently, 2 µg of RNA was reverse transcribed into complementary DNA (cDNA) using reverse transcription kits from Thermo (USA). To perform quantitative real-time PCR (qRT-PCR) analysis, we employed the ABI QuantStudio 6 Flex system from Thermo (USA). In our study, GAPDH served as the internal reference gene, ensuring accurate normalization. The primers required for qRT-PCR analysis can be found in Supplementary Table S1.

### Statistical analysis

The data were expressed as the mean ± standard deviation (SD). Statistical analysis was conducted using GraphPad Prism 7.0. Data were analyzed using two-way repeated measures ANOVA with Bonferroni's post hoc test for treatment group comparisons and one-way ANOVA with Tukey's post hoc test for group comparisons. A p-value less than 0.05 was considered statistically significant.

### Ethics approval

All experimental protocols were approved by the Medical Ethical Committee of Jiangmen Wuyi Hospital of Traditional Chinese Medicine, and all methods were carried out following ARRIVE guidelines.

## Results

### Screening of ischemic stroke-related targets

**Figure 2:**
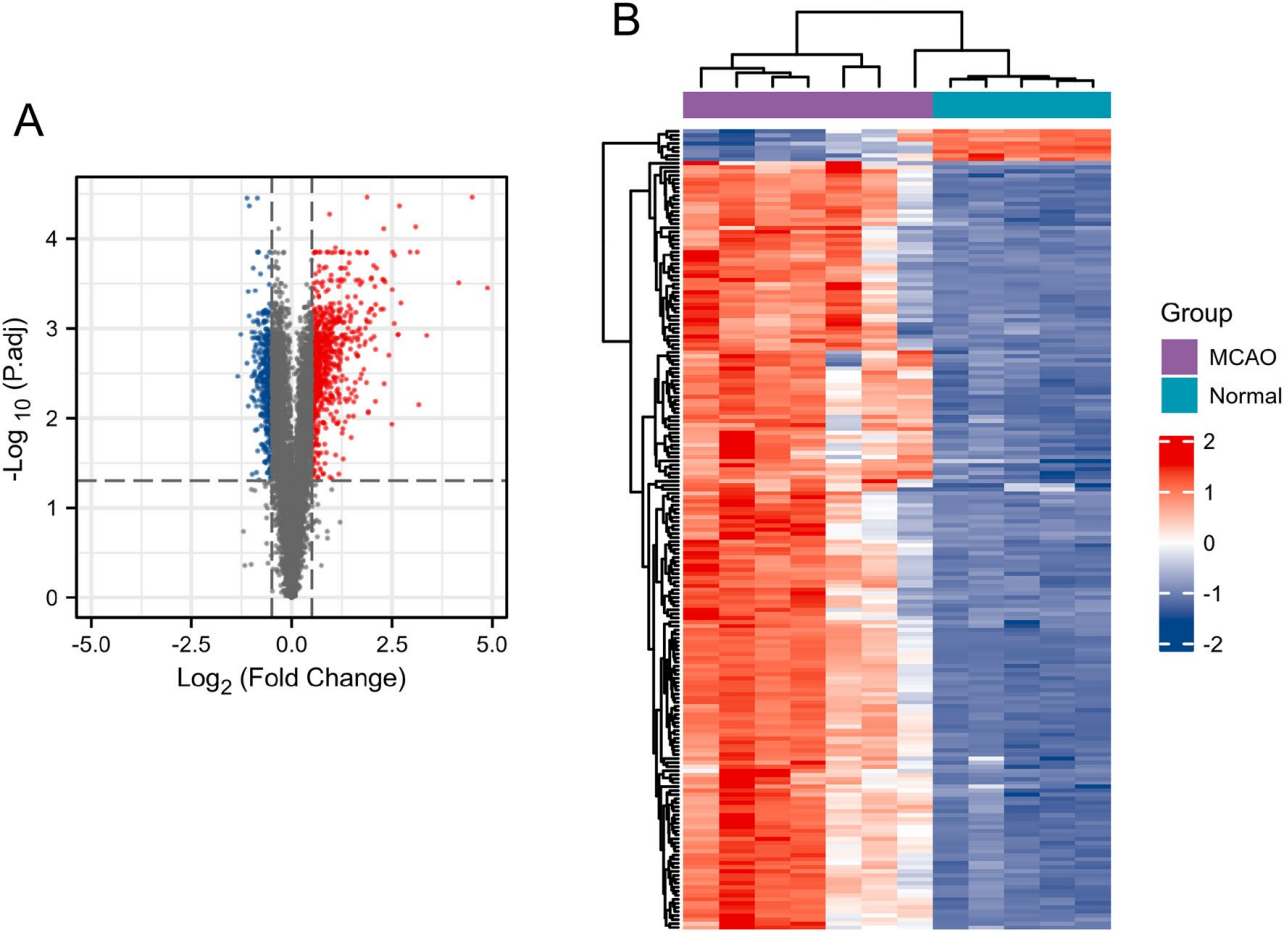
Identified differentially expressed genes (DEGs) between the normal group and the middle cerebral artery occlusion (MCAO) group. (**A**) Volcano plot of all genes in GSE97537. The red dots indicate the up-regulated genes, the blue dots indicate the down-regulated genes and the gray dots represent genes with no significant differences. (**B**) Heatmap presenting the expression levels of the top 200 DEGs in GSE97537. The purple represents the middle cerebral artery occlusion (MCAO) group, while the dark green represents the normal group. In the heatmap, red represents a significant up-regulation, while dark blue represents a significant down-regulation.

As depicted in Fig. 2A, a total of 1206 DEGs were identified when comparing the MCAO and normal groups. Among these, 766 genes were up-regulated, while 440 genes were down-regulated. The top 200 DEGs were visualized in a heatmap, as shown in Fig. 2B.

### Potential targets of PA in the treatment of ischemic stroke

**Figure 3:**
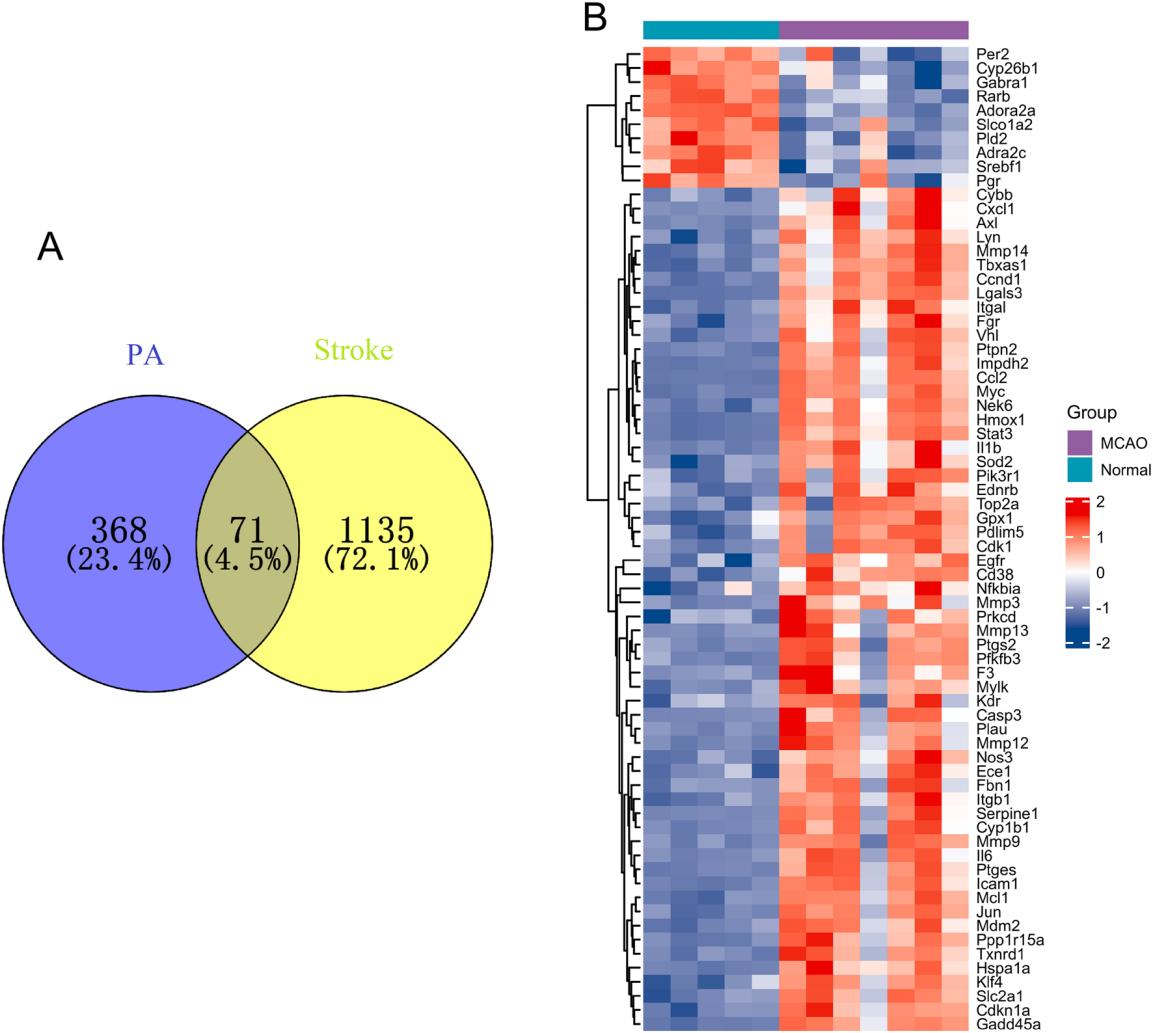
Identification of potential targets of PA in the treatment of ischemic stroke. (**A**) Venn diagram of differentially expressed genes (DEGs) in the GSE97537 database and PA-related genes. The blue area represents PA-related genes, and the yellow area represents the DEGs in the GSE97537 dataset. (**B**) Heatmap presenting the expression levels of 71 common genes. The purple represents the middle cerebral artery occlusion (MCAO) group, while the dark green represents the normal group. In the heatmap, red represents a significant up-regulation, while dark blue represents a significant down-regulation.

A total of 1206 targets related to ischemic stroke and 439 targets related to PA were inputted into the Venny online platform to determine the genes that were common to both conditions. As a result, 71 genes were found to be shared between the two (Fig. 3A). The heatmap in Fig. 3B displays the expression patterns of these shared genes.

### Functional enrichment analysis and PPI network construction of common targets

**Figure 4:**
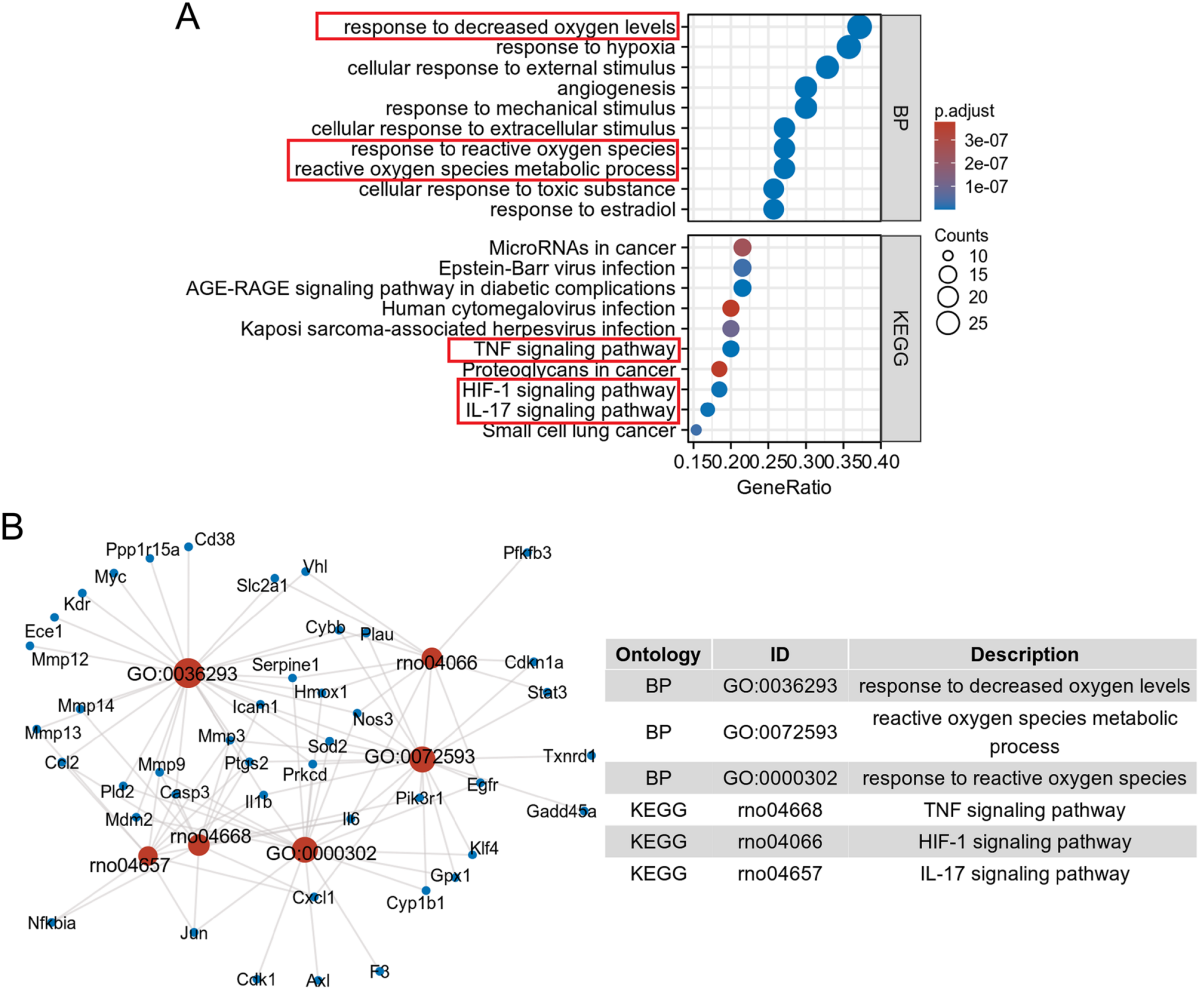
Functional enrichment analyses of common targets. (**A**) Top 10 Gene Ontology-Biological Process (GO-BP) and Kyoto Encyclopedia of Genes and Genomes (KEGG) enrichment pathways of 71 intersection genes. The colors of the bubble indicate the significance of enrichment, while size indicates the gene count. (**B**) The representative pathways of PA in the intervention of ischemic stroke. The blue nodes represent target genes and the red nodes represent the potential pathways. The size of the red nodes indicates the gene count.

To further explore the pharmacological mechanism of PA in treating ischemic stroke, we conducted GO-BP function and KEGG pathway enrichment analyses. The results of our enrichment analysis unveiled the top 10 GO-BP and KEGG signaling pathways, which were visually presented in bubble diagrams (Fig. 4A) and summarized in Table 1. Specifically, our findings demonstrated that the biological processes of PA in the treatment of ischemic stroke primarily included responses to decreased oxygen levels, reactive oxygen species, and reactive oxygen species metabolic processes (Fig. 4B). Additionally, the KEGG pathways associated with PA in ischemic stroke were mainly related to the TNF signaling pathway (Fig. S2), IL-17 signaling pathway (Fig. S3), and HIF-1 signaling pathway (Fig. S4).

**Table 1:** The top 10 GO-BP and KEGG signaling pathways.

Ontology	ID	Description	GeneRatio	Bg ratio	p value	p. adjust	q value
BP	GO:0036293	Response to decreased oxygen levels	26/70	453/17,962	5.50e − 24	1.77e − 20	8.34e − 21
BP	GO:0001666	Response to hypoxia	25/70	424/17,962	2.55e − 23	4.10e − 20	1.93e − 20
BP	GO:0071496	Cellular response to external stimulus	23/70	425/17,962	1.43e − 20	1.53e − 17	7.24e − 18
BP	GO:0009612	Response to mechanical stimulus	21/70	326/17,962	2.47e − 20	1.99e − 17	9.37e − 18
BP	GO:0072593	Reactive oxygen species metabolic process	19/70	294/17,962	1.95e − 18	1.11e − 15	5.25e − 16
BP	GO:0000302	Response to reactive oxygen species	19/70	295/17,962	2.08e − 18	1.11e − 15	5.25e − 16
BP	GO:0032355	Response to estradiol	18/70	253/17,962	3.13e − 18	1.44e − 15	6.79e − 16
BP	GO:0031668	Cellular response to extracellular stimulus	19/70	310/17,962	5.27e − 18	2.12e − 15	9.98e − 16
BP	GO:0097237	Cellular response to toxic substance	18/70	302/17,962	7.33e − 17	2.62e − 14	1.24e − 14
BP	GO:0001525	Angiogenesis	21/70	494/17,962	1.23e − 16	3.94e − 14	1.86e − 14
KEGG	rno04933	AGE-RAGE signaling pathway in diabetic complications	14/65	106/9437	8.00e − 15	1.67e − 12	8.34e − 13
KEGG	rno04668	TNF signaling pathway	13/65	116/9437	7.09e − 13	7.40e − 11	3.69e − 11
KEGG	rno04066	HIF-1 signaling pathway	12/65	120/9437	2.34e − 11	1.63e − 09	8.11e − 10
KEGG	rno04657	IL-17 signaling pathway	11/65	96/9437	3.83e − 11	2.00e − 09	9.97e − 10
KEGG	rno05169	Epstein-Barr virus infection	14/65	245/9437	8.19e − 10	2.89e − 08	1.44e − 08
KEGG	rno05222	Small cell lung cancer	10/65	96/9437	8.31e − 10	2.89e − 08	1.44e − 08
KEGG	rno05167	Kaposi sarcoma-associated herpesvirus infection	13/65	226/9437	3.30e − 09	9.84e − 08	4.91e − 08
KEGG	rno05206	MicroRNAs in cancer	14/65	296/9437	9.49e − 09	2.48e − 07	1.24e − 07
KEGG	rno05163	Human cytomegalovirus infection	13/65	259/9437	1.71e − 08	3.77e − 07	1.88e − 07
KEGG	rno05205	Proteoglycans in cancer	12/65	213/9437	1.80e − 08	3.77e − 07	1.88e − 07

**Figure 5:**
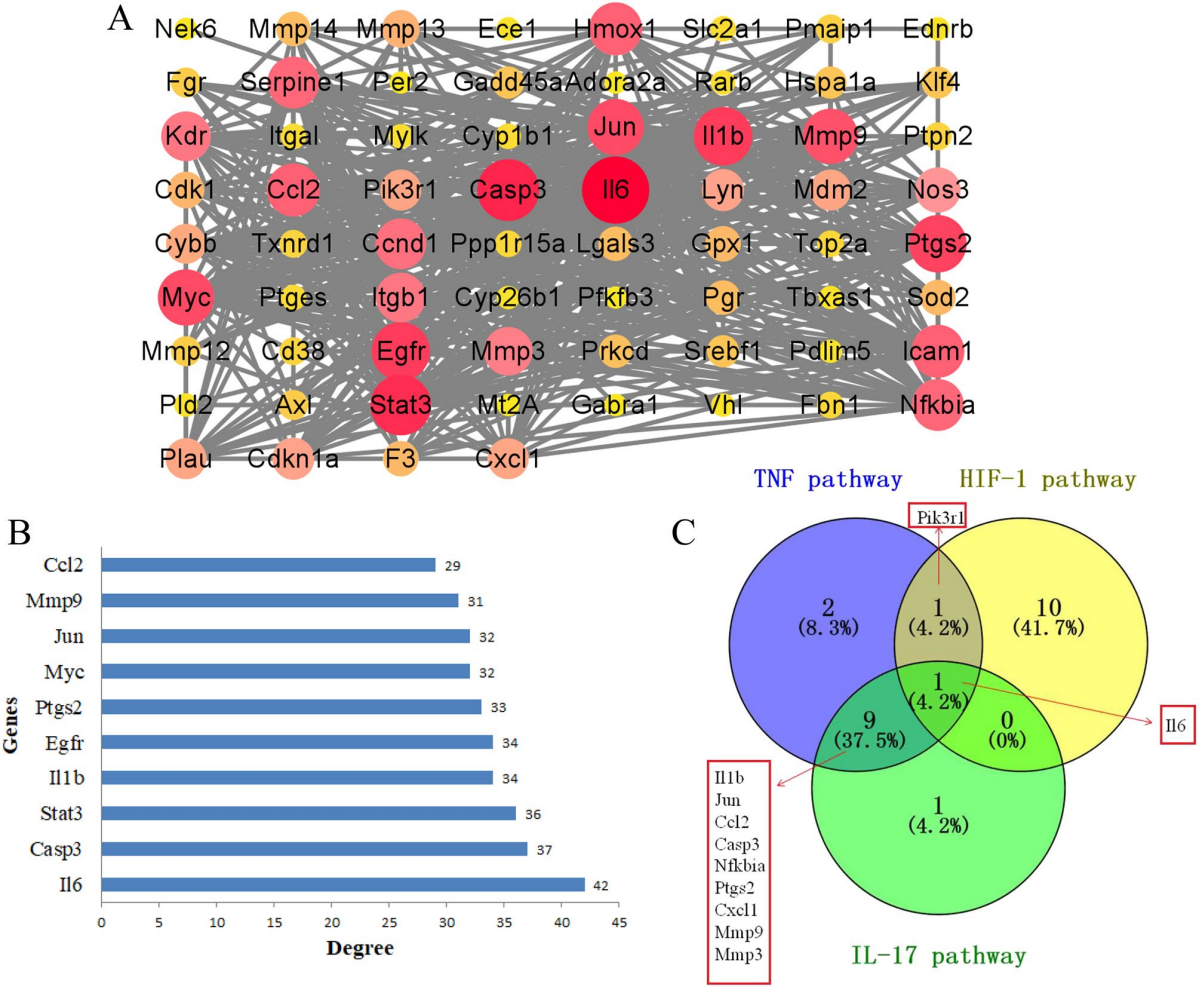
The protein–protein interaction (PPI) network of PA in the intervention of ischemic stroke. (**A**) PPI network of 71 common targets, darker nodes indicated higher importance in the network. The line indicates a direct relationship between the two genes. (**B**) Top 10 core targets in the PPI network. (**C**) Identification of important genes involved in TNF, HIF-1, and IL-17 signaling pathways. The blue, yellow, and green regions represent genes involved in the TNF, HIF-1, and IL-17 pathways, respectively.

The above 71 common targets of PA for ischemic stroke were imported into the STRING database to obtain the potential target interaction network. Then, we visualized the PPI network by using the Cytoscape software (Fig. 5A). Besides, the core targets were identified using the cytoHubba plugin of Cytoscape software based on the degree value. The top core 10 genes (Il6, Casp3, Stat3, Egfr, Il1b, Ptgs2, Myc, Jun, Mmp9, and Ccl2) were presented in Fig. 5B. In addition, the common genes (Pik3r1, Il1b, Jun, Ccl2, Casp3, Nfkbia, Ptgs2, Cxcl1, Mmp9, Mmp3, and Il6) among the TNF, HIF-1, and IL-17 signaling pathway were identified using a Venn tool (Fig. 5C).

### Neuroprotective effects of PA in MCAO-induced ischemic stroke

**Figure 6:**
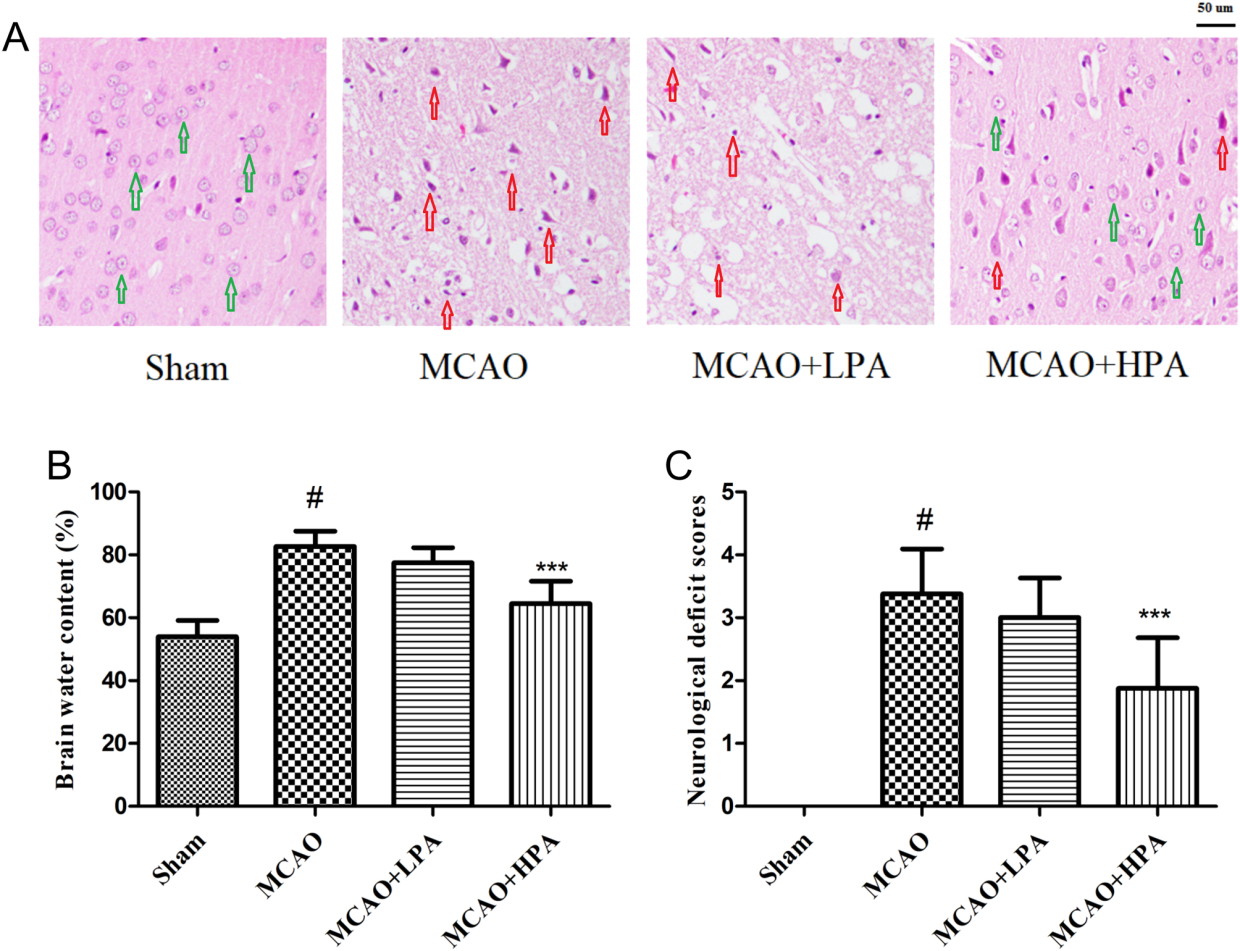
Neuroprotective effects of PA in middle cerebral artery occlusion (MCAO)-induced cerebral ischemic stroke. (**A**) Pathological changes in the brain tissues were detected by H&E staining (scale bar = 50 µm). Intact nerve cells are denoted by the green arrows, while the impaired nerve cells are highlighted by the red arrows. Brian's water content (**B**) and neurological deficit score (**C**) were in the four groups. The ratio of female to male rats was 1:1. Data were presented as mean ± standard deviation (SD), n = 6. ^#^p < 0.001, sham group vs. the MCAO group; ***p < 0.001, MCAO + HPA group vs. the MCAO group.

To examine the neuroprotective effects of PA against MCAO-induced cerebral ischemic stroke, we conducted hematoxylin–eosin staining on brain tissue sections. The results, depicted in Fig. 6A and Table 2, revealed that nerve cells in the sham group displayed normal cell morphology: visible nucleoli, compact structure, and clear cell outline. Conversely, the MCAO group exhibited disrupted and abnormal cell morphology with the disappearance of nucleoli, shrinkage or swelling of neurons, sparse nerve cells, neuronal necrobiosis, and infiltration of inflammatory cells. Notably, the histopathological changes observed in brain tissue sections from the MCAO + HPA group showed significant improvement compared to the MCAO group. However, there were no significant changes observed in the MCAO + LPA group when compared to the MCAO group.

**Table 2:** Hispathologic results of brain tissues of the different groups.

Groups	Sham	MCAO	MCAO + LPA	MCAO + HPA
Inflammatory cell infiltration	–	+++	+++	+
Neuronal necrobiosis	–	+++	+++	+

H&E stained sections scored as severe (+++), mild (+), and no lesion (–).

**Figure 7:**
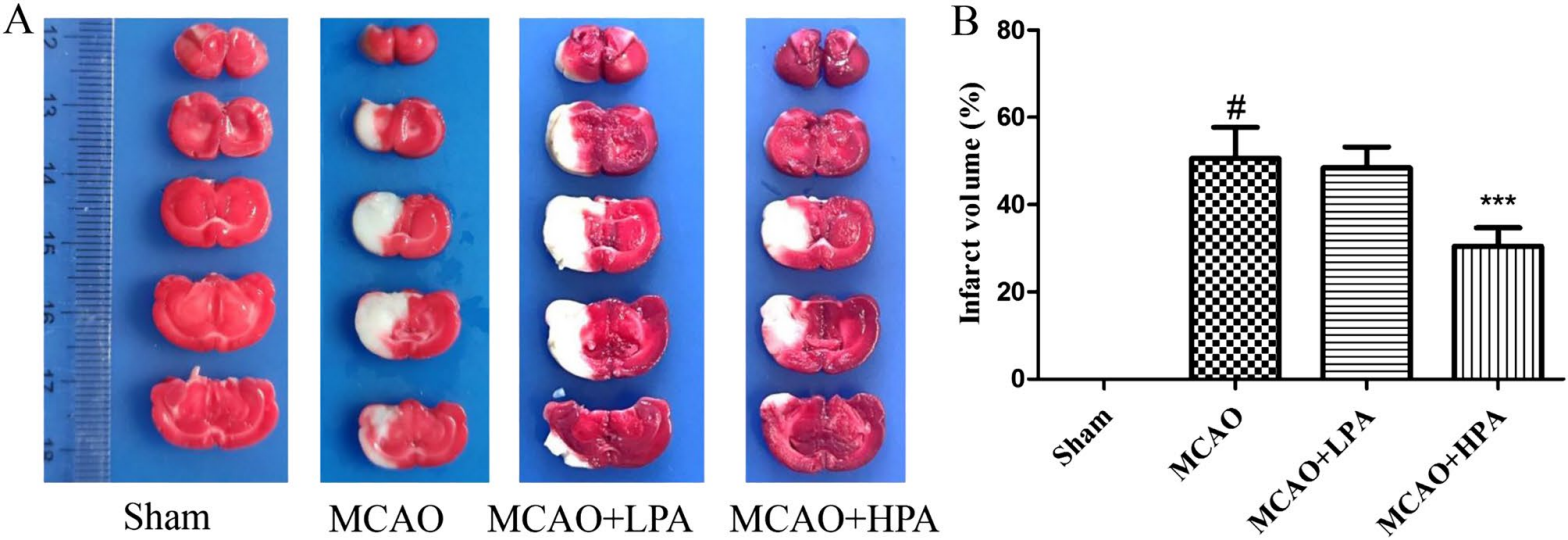
The results of TTC staining. (**A**) The four groups were assessed for infarct volume using TTC staining. (**B**) The percentage of infarct volume was determined in the four groups. The ratio of female to male rats was 1:1. Data were presented as mean ± standard deviation (SD), n = 6. ^#^p < 0.001, sham group vs. the MCAO group; ***p < 0.001, MCAO + HPA group vs. the MCAO group.

Additionally, we conducted a comparison of brain water content and neurological deficit scores among the various groups. The results, illustrated in Fig. 6B,C, revealed that both the brain water content and neurological deficit scores in the MCAO group were elevated in comparison to the sham group (p < 0.05). However, the MCAO + HPA group displayed significantly lower brain water content and neurological deficit scores compared to the MCAO group (p < 0.05). As shown in Fig. 7, the infarct volume in the MCAO group was significantly higher compared to the Sham group (p < 0.001). Nevertheless, following HPA treatment, the infarct volume showed a notable decrease (p < 0.001). Notably, no significant difference was observed between the MCAO and LPA groups (p > 0.05).

### PA relieved inflammation and oxidative stress in MCAO-induced cerebral ischemic stroke

**Figure 8:**
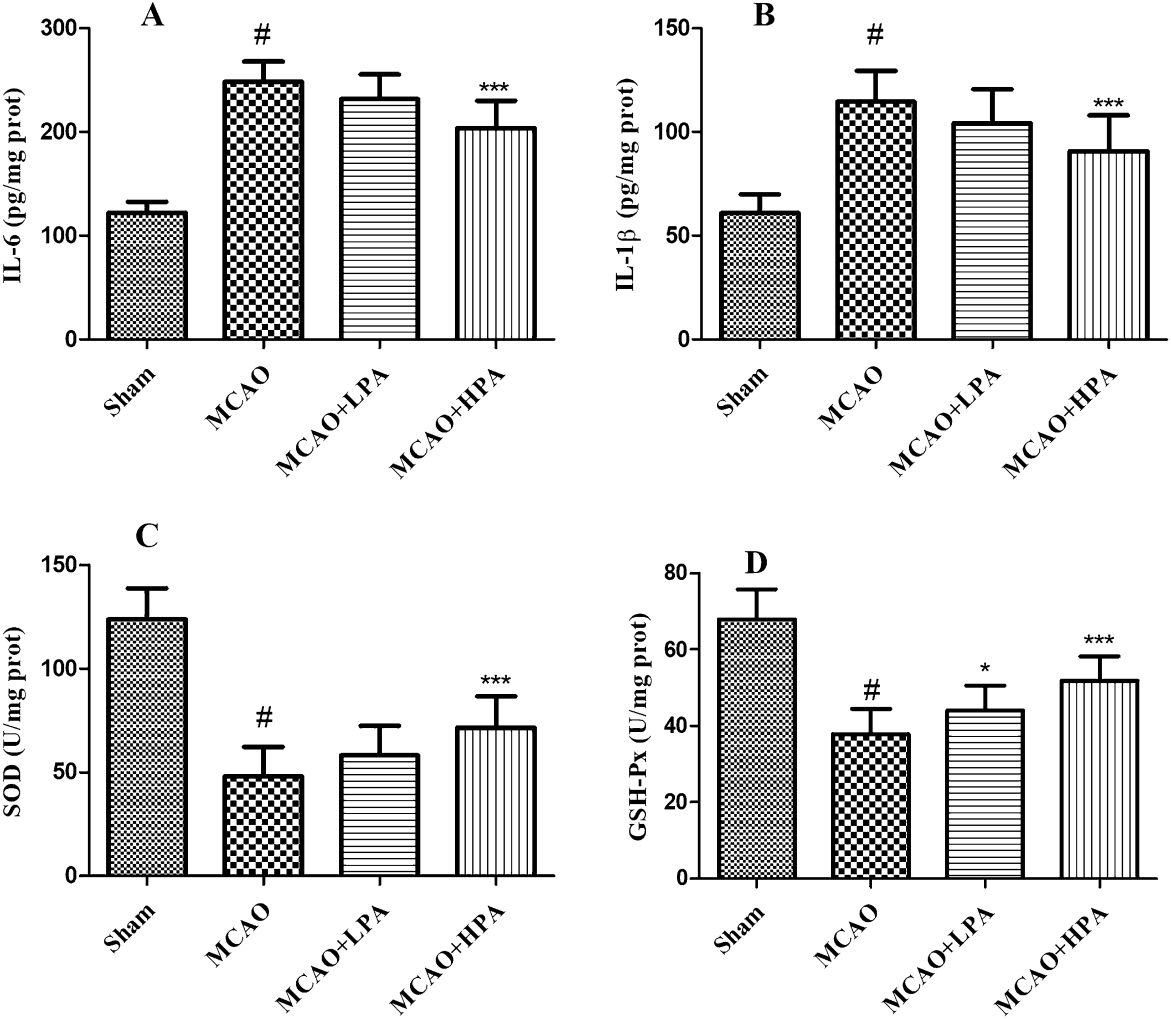
Effect of PA on inflammation and oxidative stress in middle cerebral artery occlusion (MCAO) rats. Effects of PA on the levels of IL-6 (**A**), IL-1β (**B**), SOD (**C**), and GSH-Px (**D**) in brain homogenates from rats. The ratio of female to male rats was 1:1. Data were presented as mean ± standard deviation (SD), n = 10. ^#^p < 0.001, sham group vs. the MCAO group; ***p < 0.001, MCAO + HPA group vs. the MCAO group.

In this study, we analyzed inflammation-related and oxidative stress-related markers in different groups to evaluate the effects of PA on inflammation and oxidative stress in MCAO rats. The results, illustrated in Fig. 8A,B, demonstrated a significant increase in IL-6 and IL-1β levels in the MCAO group compared to the sham group (p < 0.05). However, treatment with HPA significantly reduced IL-6 and IL-1β levels in the MCAO group (p < 0.05). Furthermore, as shown in Fig. 8C,D, SOD and GSH-Px levels were significantly decreased in the MCAO group compared to the sham group (p < 0.05). Conversely, treatment with HPA increased SOD and GSH-Px levels in the MCAO group (p < 0.05). However, there were no significant changes in the MCAO + LPA group compared to the MCAO group.

### PA-regulated core genes and genes involved in core signaling pathways in MCAO-induced cerebral ischemic stroke

**Figure 9:**
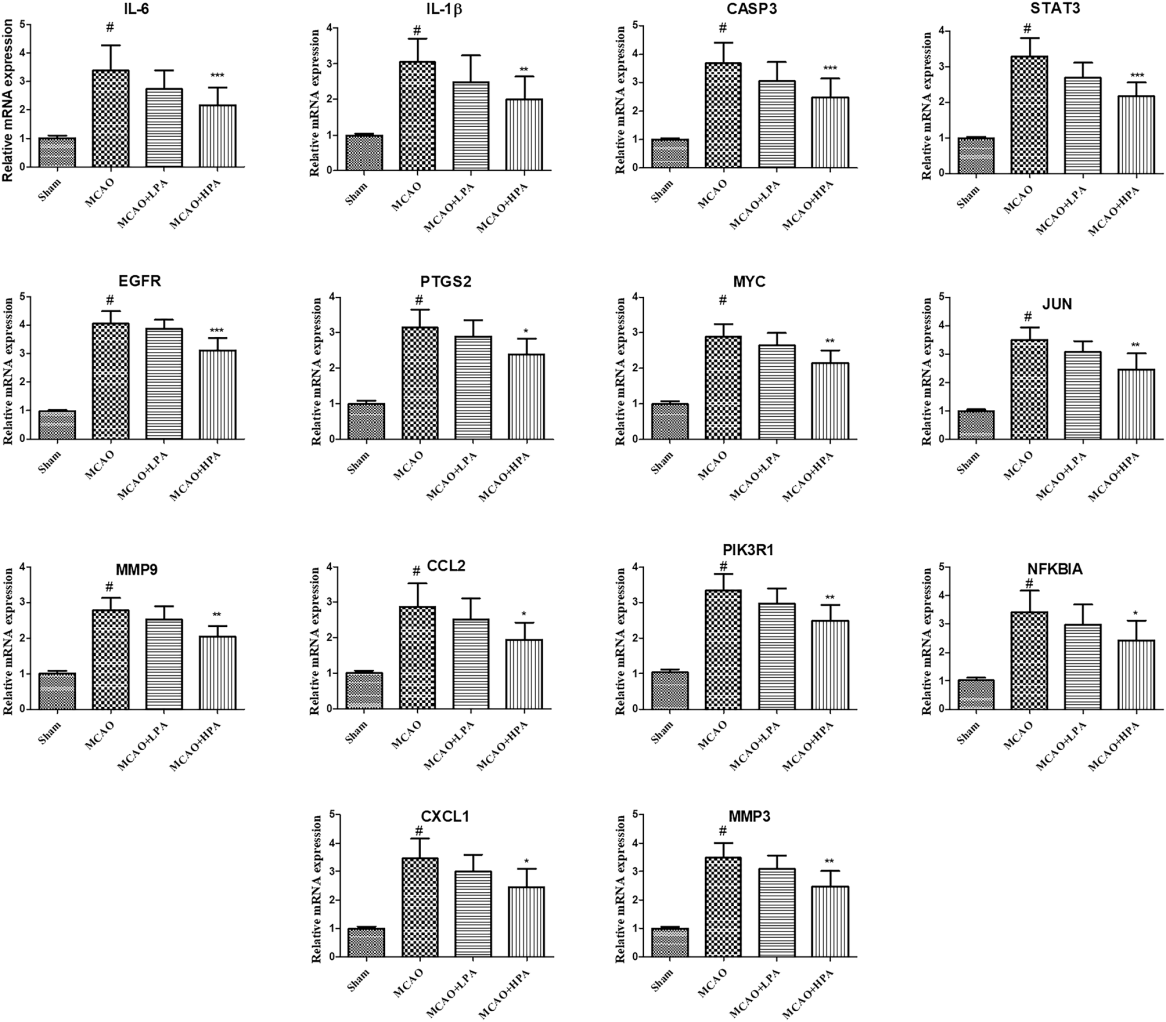
Effects of PA on the mRNA expressions of core genes and genes involved in core signaling pathways in middle cerebral artery occlusion (MCAO) rats. The mRNA levels of core genes in the brain tissues were shown by bar graphs. The ratio of female to male rats was 1:1. Data were presented as mean ± standard deviation (SD), n = 6. ^#^p < 0.001, sham group vs. the MCAO group; *p < 0.05, **p < 0.01, MCAO + HPA group vs. the MCAO group.

To confirm the findings from bioinformatics analysis, we performed qRT-PCR to analyze the mRNA expression of core targets. Figure 9 displays the results, demonstrating that the expression levels of IL-6, IL-1β, CASP3, STAT3, EGFR, PTGS2, MYC, JUN, MMP9, CCL2, PIK3R1, NFKBIA, CXCL1, and MMP3 were significantly up-regulated in the MCAO group compared to the sham group (p < 0.05). However, treatment with HPA effectively down-regulated the expression of these core genes in the MCAO group (p < 0.05).

### Cell experiments

**Figure 10:**
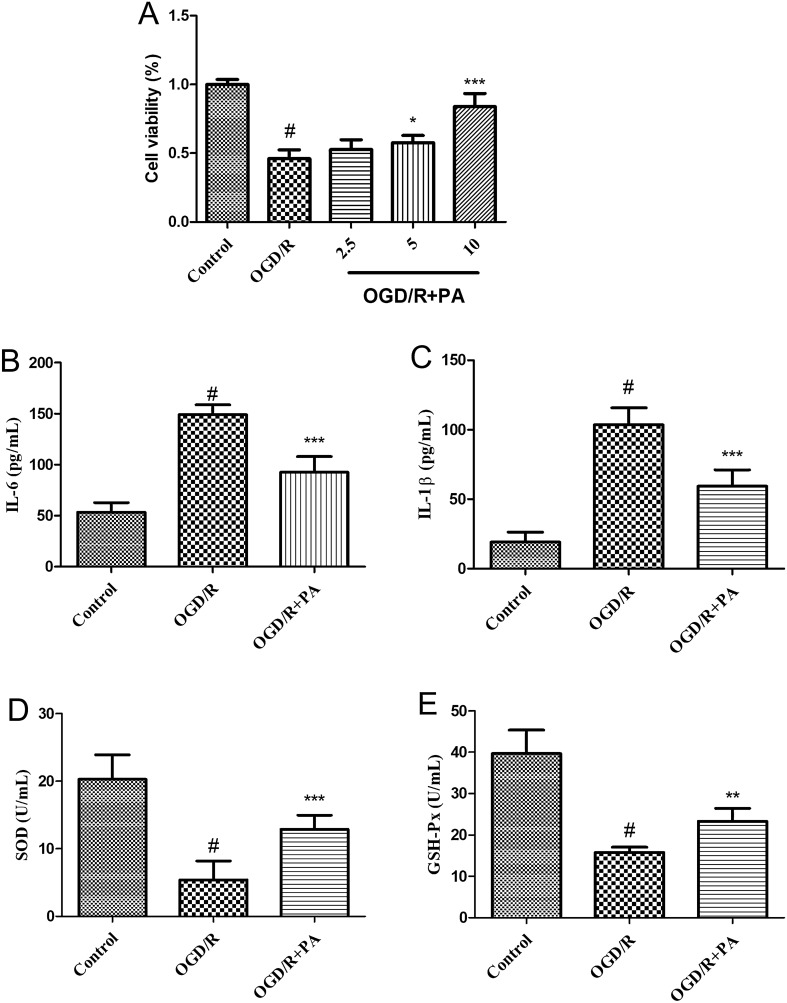
Effects of PA on the cytotoxicity, inflammation, and oxidative stress induced by OGD/R-induced in PC12 cells. (**A**) After subjecting PC12 cells to 2 h of oxygen and glucose deprivation (OGD) following 24 h of reperfusion, the cell viability was significantly enhanced by pretreating them with PA (at concentrations of 5 and 10 μg/mL) for 24 h. After 24 h of pre-exposure to PA (10 µg/mL), the concentrations of IL-6 (**B**) and IL-1β (**C**) were significantly reduced. Moreover, the activity of SOD (**D**) and GSH-Px (**D**) was enhanced following the same duration of pretreatment with PA (10 µg/mL). Data were presented as mean ± standard deviation (SD), n = 6. #p < 0.001, control group vs. the OGD/R group; *p < 0.05, **p < 0.01, ***p < 0.01, OGD/R + PA group vs. the OGD/R group.

PC12 cell viability was evaluated using the MTT assay. Figure 10A demonstrates that subjecting PC12 cells to 2 h of OGD followed by 24 h of reperfusion led to a substantial reduction (p < 0.001) in cell viability. However, pre-treating PC12 cells with varying concentrations of PA (5 and 10 μg/mL) for 24 h resulted in a significant improvement in cell viability. Following exposure of PC12 cells to OGD/R treatment, there was a significant elevation in the levels of IL-6 and IL-1β when compared to the control groups. However, pretreatment with 10 µg/mL PA remarkably attenuated the levels of IL-6 and IL-1β in OGD/R-treated PC12 cells, as depicted in Fig. 10B,C. Furthermore, following the exposure of PC12 cells to OGD/R treatment, a significant decline in the levels of SOD and GSH-Px activity was observed in comparison to the control groups. Nevertheless, the activity of SOD and GSH-Px in OGD/R-treated PC12 cells was noticeably enhanced when pretreated with 10 µg/mL PA, as depicted in Fig. 10D,E.

## Discussion

Ischemic stroke is a leading cause of disability and mortality globally, with an alarming rise in its incidence rate, particularly among younger individuals^30^. Despite the therapeutic efficacy demonstrated by various medications in the management of ischemic stroke, there are commonly encountered clinical challenges including the substantial financial burden and the potential for hemorrhagic transformation^31^. Hence, there is an urgent requirement to explore innovative approaches for treating ischemic stroke. Traditional Chinese medicine possesses a diverse array of potent medicinal compounds, offering the unique benefits of combined effects from multiple constituents, targeted actions on various pathways, and multiple therapeutic targets. PA, an active ingredient found in Paonia suffruticosa Andr., has been discovered to improve blood circulation by preventing the clumping of platelets and/or the formation of blood clots. Nevertheless, the potential mechanisms of PA against ischemic stroke have yet to be thoroughly and comprehensively investigated. To tackle this issue, our study aims to elucidate these questions through a combination of bioinformatics analysis and experimental validation.

A bioinformatics-based framework was developed in this study to explore the potential mechanisms by which PA can prevent ischemic stroke from a holistic viewpoint. A total of 71 targets associated with both PA and ischemic stroke were identified. Pathway enrichment analysis revealed that PA may exert its protective effects against ischemic stroke by primarily targeting the response to reactive oxygen species, metabolic processes related to reactive oxygen species, TNF signaling pathway, HIF-1 signaling pathway, and IL-17 signaling pathway. The regulation of pathophysiological and physiological signal transduction is largely influenced by reactive oxygen species^32^. There is evidence suggesting that the overproduction of reactive oxygen species could play a crucial part in the development of brain disorders, such as ischemic stroke^33,34^. When there is oxidative stress, the presence of reactive oxygen species can lead to the destruction of nucleic acids, proteins, and lipids through oxidative damage. As a consequence, this can have damaging effects on the structure and function of brain tissues, potentially causing cytotoxicity^35,36^. IL-17 plays a crucial role in controlling inflammation in the central nervous system. The proteins belonging to the IL-17 family have diverse effects and are highly influential in regulating both anti- and pro-inflammatory reactions. Therefore, they hold great potential as therapeutic targets for addressing ischemic stroke^37^. In the absence of any brain abnormalities, the levels of inflammatory cytokines (IL-6, IL-1β, and TNF-α) in the brain tissue are typically minimal. However, when an ischemic stroke occurs, the inflammatory response can lead to an increase in cytokines, potentially influencing phospholipid metabolism. This, in turn, may trigger the production of reactive oxygen species, ceramides, and liposomes, which have the potential to worsen brain damage^38^. In our research, we observed abnormal levels of markers related to oxidative stress and inflammation in the MCAO group, suggesting that oxidative stress and inflammation occurred following MCAO. However, when treated with HPA, the levels of IL-6 and IL-1β decreased, while the activities of SOD and GSH-Px improved. This indicates that PA effectively suppressed inflammation and oxidative stress in MCAO-induced cerebral ischemic stroke. These results align with findings from recent studies. Therefore, our results demonstrated that oxidative stress and inflammation are the potential pharmacological mechanisms by which PA in ischemic stroke treatment, which validates the bioinformatics analysis results.

The dysregulation of IL-6, an essential inflammatory marker, is strongly linked to the incidence and prognosis of ischemic stroke^39^. Additionally, IL-6 is linked to inflammation, resulting in the death of hippocampal neurons following an ischemic stroke^40^. CASP3 plays a crucial role as the primary executor and functions as a significant controller of neuronal demise during the initial phase of ischemic stroke^41^. According to a recent investigation, serum CASP3 levels were found to be elevated in cases of intracerebral hemorrhage, and this elevation was found to be associated with both clinical prognosis and severity^42^. The involvement of the JAK2/STAT3 pathway is crucial in the pathological process underlying ischemic stroke^43,44^. The increased expression of JAK2/STAT3 observed in individuals suffering from acute ischemic stroke may contribute to cellular inflammation^45^. IL-17 plays an important role in contributing to secondary damage and inflammation in post-ischemic stroke^46^. The results of our study using animal experiments showed that PA has a positive effect on cerebral ischemic stroke induced by MCAO by modulating the TNF and IL-17 pathways. These findings provide support for the predicted mechanism of PA in treating ischemic stroke using systems bioinformatics. Additionally, our results unveil the potential therapeutic mechanism of PA for ischemic stroke and provide a scientific foundation for future research.

Nonetheless, there are several limitations to our study. Our research exclusively concentrated on examining the implications of oxidative stress and inflammation in MCAO-induced cerebral ischemic stroke. We must delve into other pathophysiological processes such as excitotoxicity, autophagy, apoptosis, and inflammasome to gain a comprehensive understanding.

## Conclusion

To summarize, bioinformatics analysis and animal experiments were conducted to discover potential pathways and targets of PA in treating ischemic stroke. Our study revealed that the potential mechanisms by which PA prevents ischemic stroke involve oxidative stress and inflammation. These findings offer a fresh perspective on the pharmacological mechanisms of PA in preventing ischemic stroke, thus promoting further exploration, application, and development of traditional Chinese medicine.

### Supplementary Information

Supplementary Information.

## Data Availability

All of the data in this study are available from Zhiyan Wu.
