# Supplementary material for: Bioinformatics analysis and experimental validation revealed that Paeoniflorigenone effectively mitigates cerebral ischemic stroke by suppressing oxidative stress and inflammation
Source: Sci Rep. 2024 Mar 7;14:5580. doi: 10.1038/s41598-024-55041-5 (PMC10918059; doi:10.1038/s41598-024-55041-5)
Supplement: Supplementary file 1 — Supplementary Information. [file 41598_2024_55041_MOESM1_ESM.docx]

**Table S1.** Sequences of primer used in quantitative real-time PCR.

| Gene | Forward primer (5' to 3') | Reverse primer (5' to 3') |
| --- | --- | --- |
| Il6 | TGGTGATAAATCCCGATGAAG | GGCACTGAAACTCCTGGTCT |
| Il1β | TGAAATGCCACCTTTTGACAG | CCACAGCCACAATGAGTGATAC |
| Casp3 | CAACAACGAAACCTCCGTGG | CTGCTCCTTTTGCTGTGATC |
| Stat3 | TACCAGCAAAATCAGGTTGCT | ACATCCCCAGA GTCCTTATCAA |
| Egfr | ACCTGCGTGAAGAAGTGTCC | CGTTACACACTTTGCGGCAAGG |
| Ptgs2 | TCAGAAGCGAGGACCTGGG | TACACCTCTCCACCGATGAC |
| Myc | ATGCCCCTCAACGTGAACTTC | GTCGCAGATGAAATAGGGCTG |
| Jun | TCCAAGTGCCGAAAAAGGAAG | CGAGTTCTGAGCTTTCAAGGT |
| Mmp9 | GATCCCCAGAGCGTTACTCG | GTTGTGGAAACTCACACGCC |
| Ccl2 | TAAAAACCTGGATCGGAACCAAA | GCATTAGCTTCAGATTTACGGGT |
| Pik3r1 | CGAAAACACAGAAGACCAATACTCA | TCCCTCGCAATAGGTTCTCG |
| Nfkbia | GCCTAGCCCCGAGCATTC | AATGATCTGTTTCCCCAAATTTCA |
| Cxcl1 | GGCAGGGATTCACTTCAAGA | ATCTTGAGCTCGGCAGTGTT |
| Mmp3 | AGTGTGGATTCTGCCATTG | GAGTTCCATAGAGGGACTGAATAC |
| GAPDH | TGAAGGTCGGAGTCAACGG | TGGAAGATGGTGATGGGAT |


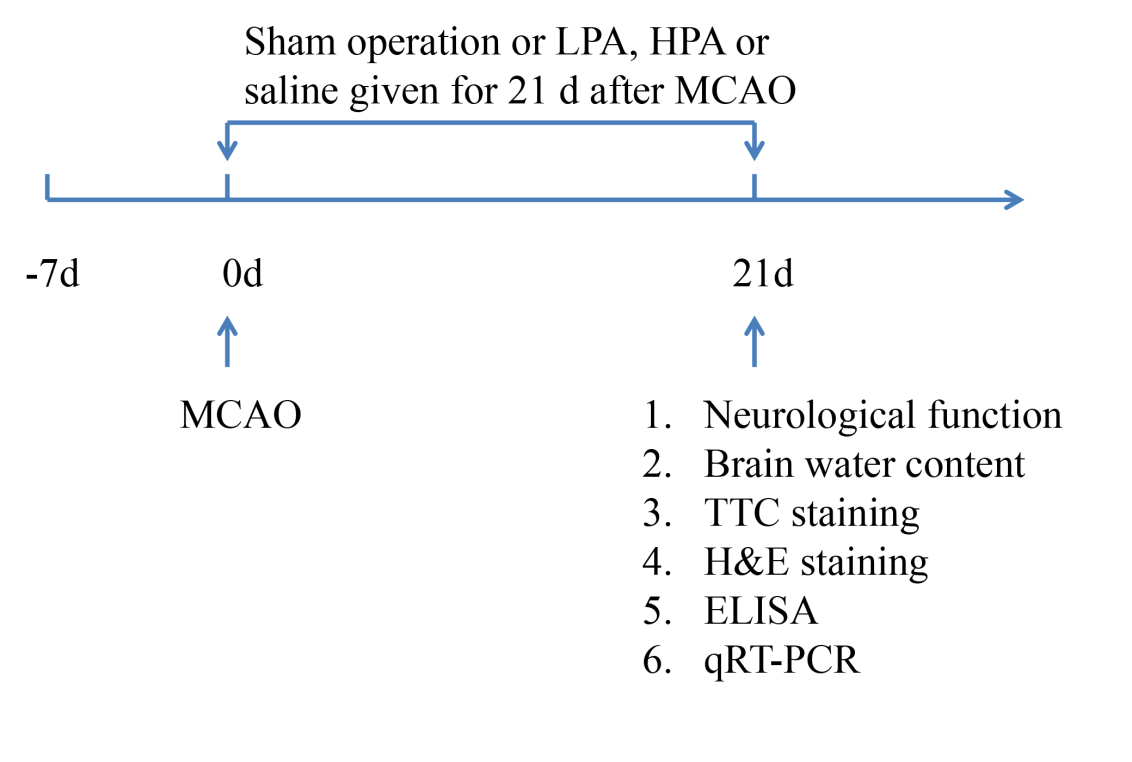


Figure S1 The schedule and design of the experiment.


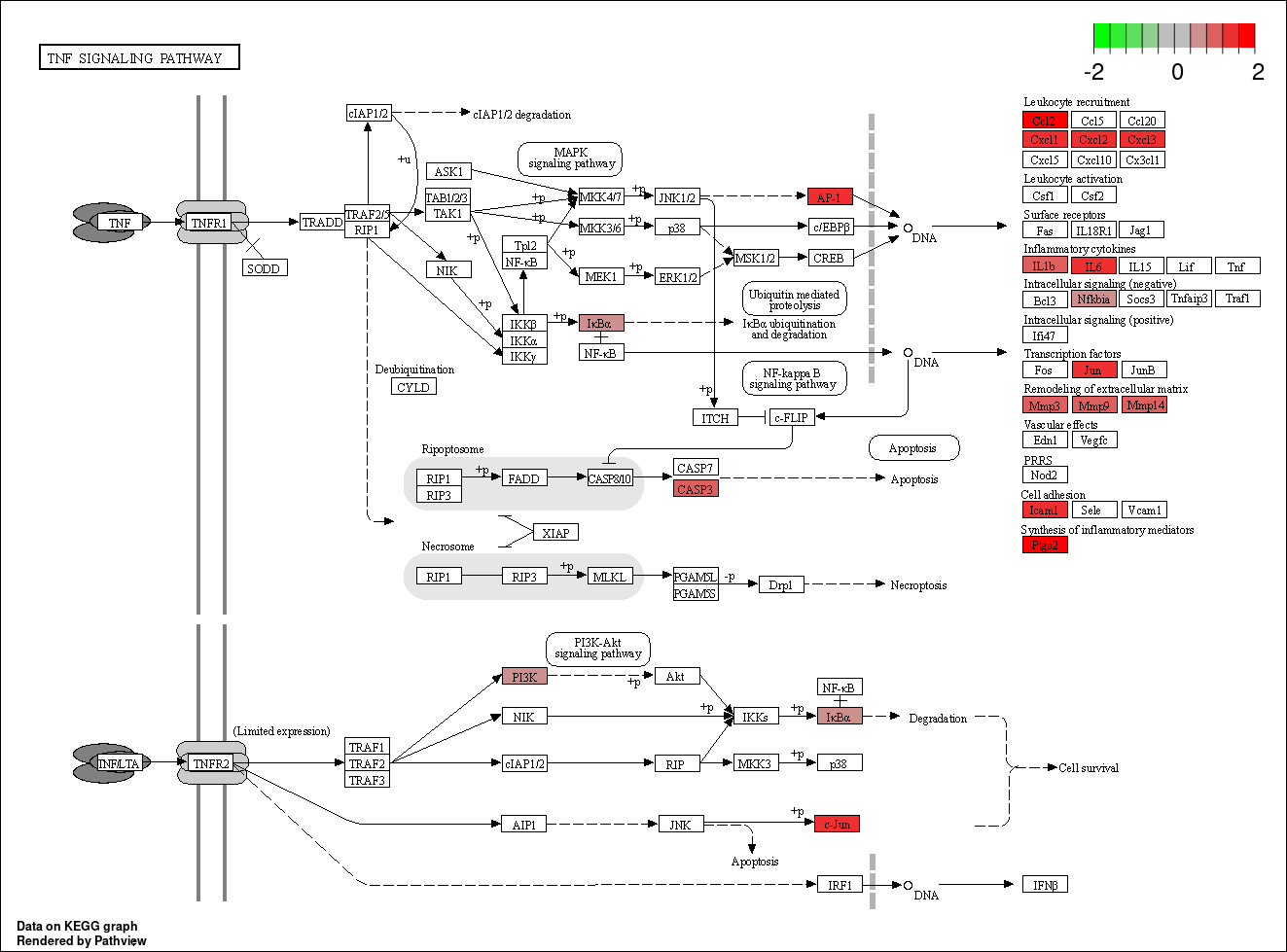


Figure S2 Therapeutic effects of PA against MCAO involved in TNF signaling pathway. The red rectangle indicates the genes associated with the PPI network.


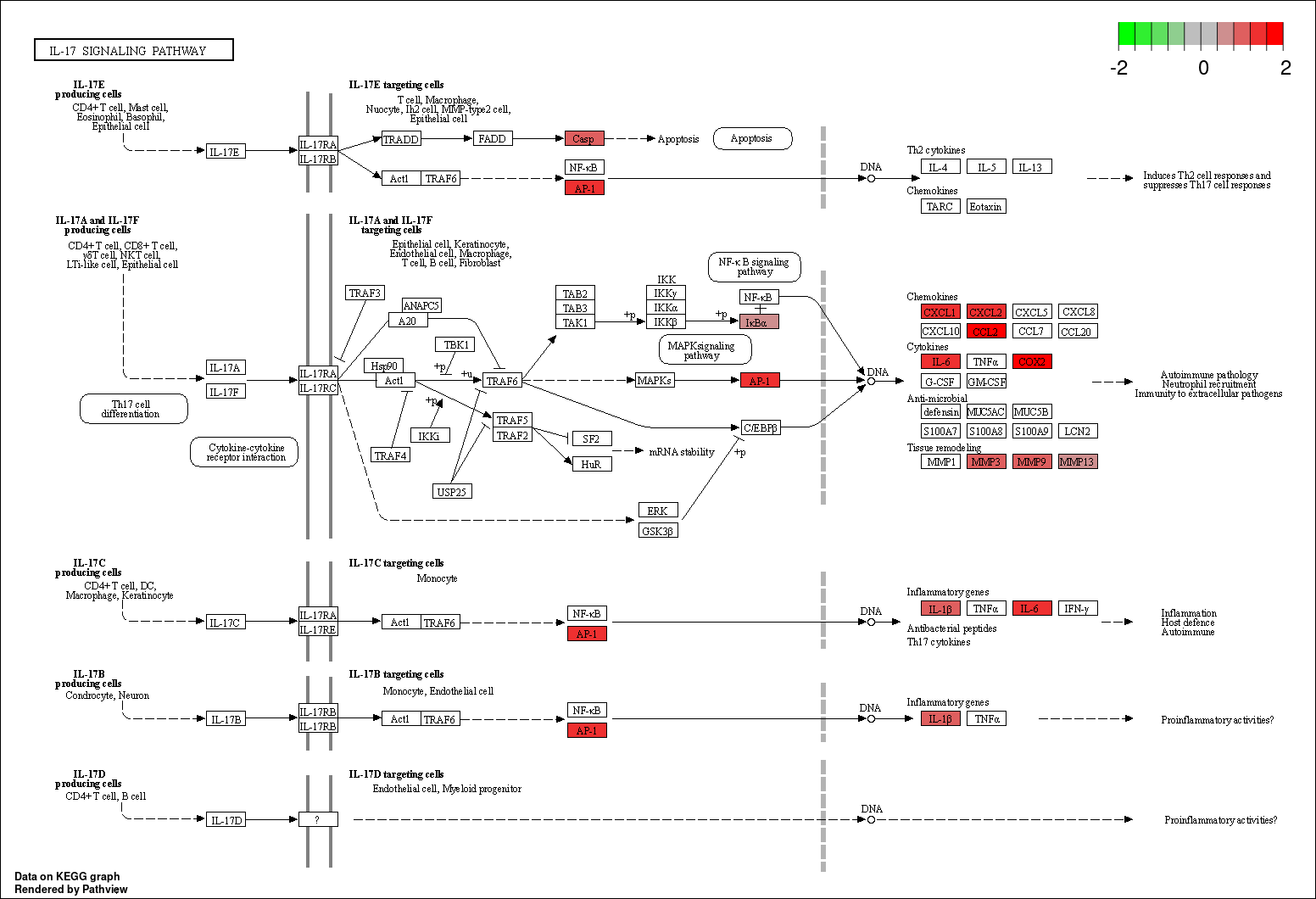


Figure S3 Therapeutic effects of PA against MCAO involved in IL-17 signaling pathway. The red rectangle indicates the genes associated with the PPI network.


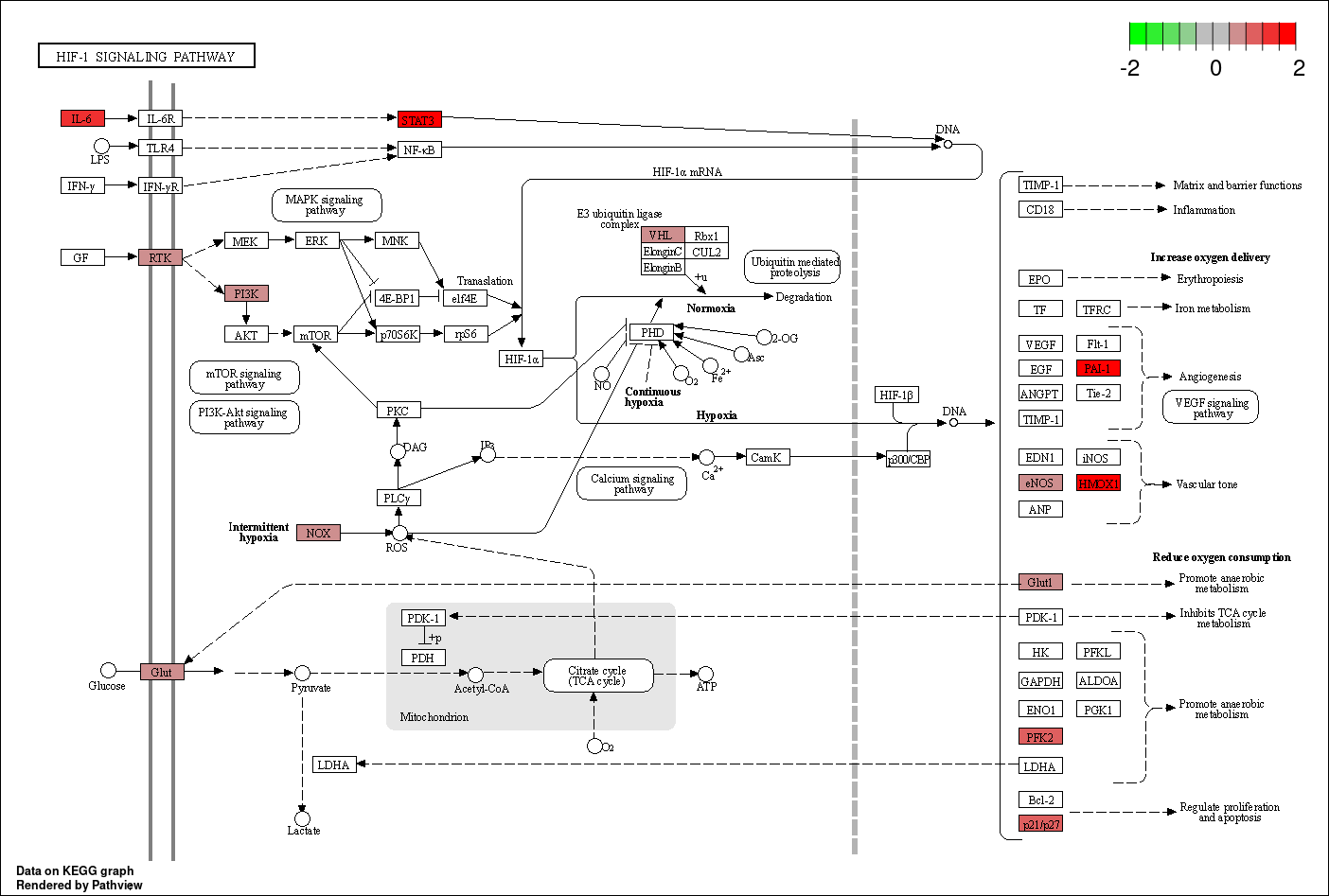


Figure S4 Therapeutic effects of PA against MCAO involved in HIF-1 signaling pathway. The red rectangle indicates the genes associated with the PPI network.
